# Bibliographical

**Published:** 1876-04

**Authors:** 


### BIBLIOGRAPHICAL.

*A System of Midwifery, Including the Diseases of Pregnancy and the Puerperal State, by* WM. LIESHMAN, M.D., *Regius Professor of Midwifery in the University of Glasgow, Physician to the University Lying-in-Hospital, Fellow (and Late Vice-President) of the Obstetrical Society of London, etc. etc.; second American from the second and Revised English Edition, with Additions of* JOHN S. PARRY, M.D., *Obstetrician to the Philadelphia Hospital, Vice-President of the Obstetrical Society of Philadelphia, etc.* Henry C. Lea, Publisher—1875.

*Lieshman's System of Midwifery* has been adopted by a number of our medical schools as a text-book, a fact which, viewed in connection with the simultaneous rapid exhaustion of the previous editions, both English and American, indicates its great value, and strongly commends it to the profession everywhere.

*A Treatise On the Diseases of Infancy and Childhood: By* I. LEWIS SMITH, M.D., *Physician to the New York Infants' Hospital, Catholic Foundling Asylum, to Protestant Infant Asylum, Clinical Lecturer on Diseases of Children in Bellevue Hospital Medical College; Third Edition, Enlarged and Thoroughly Revised, with illustrations.* Philadelphia, Henry C. Lea, 1876.

This work, already well established, again comes to the profession in a new and enlarged volume of 700 pages, containing new and important practical additions. The connection of the author with several large institutions in the city of New York gives him unusual facilities for the clinical study of the peculiar and different class of diseases pertaining to children, of which he has well availed himself, as is evident from this admirable edition of his work. We note a chapter on the eruptive disease called, in the British Authorities, *rotheln*, also one on cerebro-spinal fever, with new and practical suggestions on diphtheria and other affections.

*A Treatise on Surgery. Its Principles and Practice. By* T. HOLMES, *Cantab, Surgeon to St. George's Hospital, with* 411 *il-*

*lustrations, chiefly by* DR. WESTMACOTT. Philadelphia: Henry C. Lea, Publisher, pp. 960.

This is a new, and unquestionably a very valuable work on Surgery. The author has aimed, and we think very successfully, to represent the present condition of British Surgery. The style of the work is terse, condensed and practical, freed from tiresome and minute details of pathology, and hence is acceptable, both as a text-book for students and to the daily wants of the busy practitioner.

Reference is made, also, to American and Continental Surgery, and, so far as we are able to judge from a somewhat cursory glance at its contents, it embraces all topics included under the title "*surgical*," with interesting chapters on diseases of the eye, ear, skin, etc. We note that reference is made to Esmarch's bandage and Dieulafoy's aspirator, indicating that the work is brought down to the latest discoveries and improvements in the art. We hesitate not to commend it to the profession as a work of great merit.

*Extra-Uterine Pregnancy. Its Causes, Species, Pathological Anatomy, Clinical History, Diagnosis, Prognosis and Treatment. By* JOHN S. PARRY, M.D., *Obstetrician to Philadelphia Hospital, Physician for Diseases of Women in Presbyterian Hospital, Fellow of the College of Physicians, Philadelphia, etc.* Philadelphia: Book, cloth, pp. 276: Henry C. Lea.

This work is based upon an analysis of five hundred cases of extra-uterine pregnancy, and must prove novel, instructive, and interesting to the professional reader. Those who have given no special attention to this subject will be surprised at the classification of the varied localities in which an extra-uterine child may be developed. We have not space to detail them, but recommend our readers to procure the work. It will repay perusal.

